# Designing magnetic superlattices that are composed of single domain nanomagnets

**DOI:** 10.3762/bjnano.5.109

**Published:** 2014-07-03

**Authors:** Derek M Forrester, Feodor V Kusmartsev, Endre Kovács

**Affiliations:** 1 Department of Physics, School of Science, Loughborough University, Leicestershire, LE11 3TU United Kingdom; 2 Department of Physics, University of Miskolc, H-3515 Miskolc, Hungary

**Keywords:** hysteresis, nanoparticles, magnetic phases, superlattices

## Abstract

**Background:** The complex nature of the magnetic interactions between any number of nanosized elements of a magnetic superlattice can be described by the generic behavior that is presented here. The hysteresis characteristics of interacting elliptical nanomagnets are described by a quasi-static method that identifies the critical boundaries between magnetic phases. A full dynamical analysis is conducted in complement to this and the deviations from the quasi-static analysis are highlighted. Each phase is defined by the configuration of the magnetic moments of the chain of single domain nanomagnets and correspondingly the existence of parallel, anti-parallel and canting average magnetization states.

**Results:** We give examples of the phase diagrams in terms of anisotropy and coupling strength for two, three and four magnetic layers. Each phase diagrams character is defined by the shape of the magnetic hysteresis profile for a system in an applied magnetic field. We present the analytical solutions that enable one to define the “phase” boundaries between the emergence of spin-flop, anti-parallel and parallel configurations. The shape of the hysteresis profile is a function of the coupling strength between the nanomagnets and examples are given of how it dictates a systems magnetic response. Many different paths between metastable states can exist and this can lead to instabilities and fluctuations in the magnetization.

**Conclusion:** With these phase diagrams one can find the most stable magnetic configurations against perturbations so as to create magnetic devices. On the other hand, one may require a magnetic system that can easily be switched between phases, and so one can use the information herein to design superlattices of the required shape and character by choosing parameters close to the phase boundaries. This work will be useful when designing future spintronic devices, especially those manipulating the properties of CoFeB compounds.

## Introduction

The development of highly ordered superlattices is of significant importance from a practical perspective as well as for discovering new collective properties. The cumulative magnetic effects associated with the interaction of many nanomagnets can be witnessed as giving rise to unique-phase transitional behaviors. This is especially true for thin elliptical cylinder nanomagnets that have high shape anisotropies [1]. A nanomagnet with high shape anisotropy must have some kind of modulation in order to reduce the height of the anisotropy energy barrier. This is typically done through doping in order to reduce the saturation magnetization of the nanomagnet. In recent years amorphous ferromagnetic materials have been successfully doped (with, for example, vanadium or chromium [2]) to lower the saturation magnetization of the compound. In doing so the energy barrier between stable states of the magnetization remains at a surmountable level. This is of high significance for designing magnetic logic elements and magnetic sensors because changing the geometry of the nanomagnet and levels of doping allows one to change the magnetic properties in a controllable way. Superlattices with magnetic elements of amorphous magnetic alloys, such as Co_60_Fe_20_B_20_ [3] and Fe_65_Co_35_B_12_ [4], have been developed due to their excellent room temperature magnetoresistance properties. Indeed, these bromide alloys are prime candidates for creating functional magnetic field sensors and magnetic random access memory devices. Recent work has even focused on controlling the nucleation and propagation of topological magnetic solitons through CoFeB/Ru superlattice stacks [5]. With their excellent magnetic properties and soft magnetic character, amorphous magnetic materials will continue to be used in future devices. Thus, we investigate the generic magnetic response of nanomagnets that are composed of amorphous magnetic materials that have low random anisotropy.

In this work we study the magnetic response of linear arrays of nanomagnets to an oscillating magnetic field that is applied at a slight angle from parallel to the longest axis of an individual nanomagnet. A small perturbing magnetic field is also introduced that enables the system to locate the metastable states that exist in the energy landscape. The size of the shape anisotropy energy barrier is a function of the saturation magnetization, the geometry of the nanomagnet and its demagnetization factors.

These demagnetization factors, *N_x_*_,_*_y_*_,_*_z_*, are defined by length scales associated with the *x*, *y* and *z* axes of a nanomagnet. To a reasonable degree of accuracy, given the high accuracy of modern fabrication technologies, each of the *N* magnetic layers in the superlattice can be taken as having the same demagnetization factors. Each nanomagnet in the system, each given an index *i* = 1,2...,*N*, has lengths *l_x_*_,_*_y_*_,_*_z_*. The nanomagnets in the superlattice, each of which is a thin elliptical cylinder of volume *V*, have dimensionless magnetization vectors associated with them: **m***_i_* = (*m_xi_*,*m_yi_*,*m_zi_*) = (cosφ*_i_* sinθ*_i_*,sinφ*_i_* sinθ*_i_*,cosθ*_i_*). Here, we focus primarily upon structures that have antiferromagnetic coupling between nanomagnets. Ferromagnetic coupling invariably leads to magnetization hysteresis profiles that depict parallel magnetizations of equal magnitude and direction for all the constituent nanomagnets. In artificial superlattice structures the thickness of the interlayer is manipulated to enforce an antiferromagnetic coupling. In all superlattices the lattice period of the layers is of crucial importance to defining its purpose and physical properties [6–7].

The properties of the spacer layers (composed of MgO or Ru, for example) between the nanomagnets, and the resulting interfacial exchange coupling, usually leads to magnetization saturation fields in superlattices that are smaller than those of individual magnetic layers [8]. The coupling energy is a function of the thickness of the interlayer. Experimentally, it has been found that creating a ruthenium interlayer of 1.2–1.5 nm between CoFeB nanomagnets has the consequence of generating an antiferromagnetic coupling, whereas with a larger thickness ferromagnetic coupling will ensue [9]. We investigate the changes in the hysteresis profiles that become apparent in systems of antiferromagnetically coupled nanomagnets when the coupling strength is altered.

It is important to note that the interaction of magnetic disks in a stack may have a dipolar character. For example, if we consider two small elliptical magnetic particles in a vacuum, separated by large distances from each other so that each particle will have a large magnetic moment, then these particles will primarily have only dipolar interaction. However, when the distance between these particles decreases, the inter-particle interaction will be modified. The latter depends on the specific media separating the particles. If this is a metal then there appears the RKKY interaction, which is mediated by the electrons of the surrounding metal. Depending on the distance between particles it may have both antiferromagnetic and ferromagnetic character. However if the media between the particles is not metallic and say, a dielectric, then at each magnetic particle there may be induced higher multipoles which add additional contributions into the overall interaction.

Thus, the resulting interaction between the particles depends on the relative interaction between them, so that the shape anisotropy will play a key role. Recently, Serantes et al. [10] produced a methodology that is based upon a very powerful computational Monte Carlo technique to study the magnetic ordering in a system of dipolarly interacting magnetic nanoparticles distributed along 1D chains. They studied in detail the very interesting issue of the interplay between the nanoparticles magnetostatic dipolar interaction and magnetic anisotropy [10]. The separation distance for dipole–dipole interactions also determines whether the system has an affinity for an antiferromagnetic or ferromagnetic interaction. Therefore, in their work two different cases, non- and collinear, are considered [10]. So, in the present paper we have considered the case when the particles are packed into the stack. Here the magnetic moments are oriented perpendicular to the stack (or chain of particles). Due to this form of construction the inter-particle dipolar interaction has primarily the antiferromagnetic character (which, in the next section, we will describe by the constant of inter-particles interaction, *J*).

Thus, our approach is consistent with the studies [10–13], which found by computational Monte Carlo techniques that the magnetization of the dipolarly interacting magnetic nanoparticles is well described by the Curie–Weiss law. Moreover, Serantes et al. [10] obtained in the mean-field approximation that even non-interacting particles are described by the Curie–Weiss law where the critical parameter, an ordering temperature, stands for the antiferromagnetic order. Because of that fact the general conclusion of these papers was that a mean-field treatment is not adequate to study magnetic nanoparticle systems [10–13]. Therefore, in the present paper we developed a microscopic approach which is based on considering the dynamical behavior of magnetic nanoparticles with the use of coupled micromagnetic equations. The results we obtain are consistent both with a macroscopic Curie–Weiss type fitting and the microscopic calculations of Serantes and co-workers [10], which point to the primary importance of the shape anisotropy of nanoparticles and the resulting magnetic properties of these systems.

## Results

### The superlattice magnetic energies and magnetic fields

By lowering the saturation magnetization (*M_s_*) of magnetic compounds such as CoFeB by doping [2] one can extend the elongation of each nanomagnet without creating too high an energy barrier between the stable states of the superlattice [14–15]. This is important for creating magnetic logic or memory devices, so as to enable switching between different orientations of the magnetic moments in moderate magnetic fields. The height of the energy barrier is reduced by doping and in order to compensate for the resulting off-set in the height of the barrier a higher aspect ratio nanomagnet has to be designed. The shape anisotropy is given by [15],

[1]
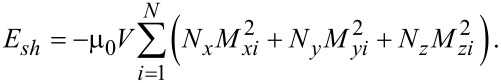


In this work we define the longest axis of each nanomagnet to be in the *x*-direction and so the smallest shape anisotropy is associated with this *x*-coordinate (tending to zero the more stretched a magnet becomes). A superlattice composed of interspersed nanomagnets can be created by balancing the aforementioned energy concerns to create a robust system against thermal instabilities (shaped as an elliptical stack). The protection of magnetic information can be achieved due to the increase in magnetic volume and the reduced switching current density [16]. The energy equations for the system of *N* nanomagnets are,

[2]
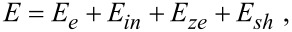


where the interaction energies between the end and next nearest to the end nanomagnets (one nearest neighbor) are given by *E_e_*

[3]
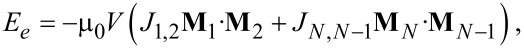


the inner interaction energies (two nearest neighbors) *E_in_* are,

[4]
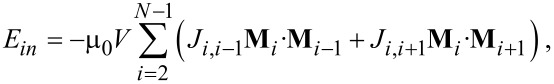


and the Zeeman energies *E_ze_* are

[5]
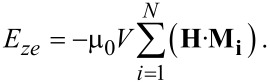


Later, we will define all the coupling energies equal, with *J* being a dimensionless coupling parameter. When *J* is negative the coupling is antiferromagnetic for certain interlayer thicknesses. The nature of the coupling changes according to the mechanisms of Ruderman–Kittel–Kasuya–Yosida (RKKY) interaction theory [17–19]. The field applied to the superlattices is **H** = *M_s_***h** = *M_s_* {*h_x_*,*h_y_*,*h_z_*} (with *M_s_* = 0.5 × 10^6^ A/m) and we reintroduce the notation {*e*,*a*,*b*} = 2{*N_x_*,*N_y_*,*N_z_*} from [15]. Now, the dynamical equations for the magnetization are derived from the Landau–Lifshitz–Gilbert (LLG) equation ([15,20–21]),

[6]
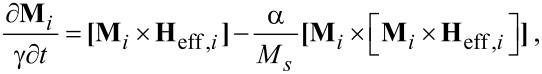


where **H**_eff,_*_i_* is an effective field and γ is the gyromagnetic ratio. In the last term, the Gilbert damping, with damping parameter α, is incorporated into the model. Equation 6 is expanded as in [22] to find the evolution of the magnetization angles in each nanomagnet. A resulting dimensionless form is found by dividing by *M_s_*^2^ and writing time as *t* = τ/γ *M_s_*. The dimensionless parameters used here (*J*, *e*, *a*, *b*, and *h_x_*_,_*_y_*_,_*_z_*), have also been divided by *N_x_* throughout [15]. The magnetizations are defined through *∂*φ*_i_*/*∂*τ and *∂*θ*_i_*/*∂*τ. The resulting 2*N* coupled first-order differential equations are then solved with a numerical algorithm based upon adaptive Runge–Kutta–Cash–Karp techniques. The oscillating magnetic field is directed along the *x*-axis of the superlattice, i.e., the longest axis, with a frequency of 1 GHz.

### The response of nanomagnets to an applied magnetic field

Throughout we use the damping parameter equal to α = 0.01 and a large value of *b* (about 390) to confine the magnetic moments to move in the *x*–*y*-plane. We investigated nanomagnets with semi-major to semi-minor elliptical cross-sections of *l_x_*/*l_y_* ≈ 10. The external magnetic field in the *x* and *y* components,

[7]
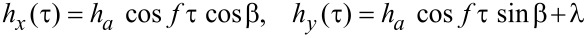


is applied with frequency *f*_applied_ = *f*γ*M_s_* and amplitude *H_a_* = *M_s_h_a_*. The angle of deviation between the applied field and the *x*-axis of the superlattice is β, and λ is a small time-dependent perturbation in *h_y_* with amplitude 0.02 (the perturbation has its origin in a thermally assisted magnetization reversal, as discussed on page 6 of [15] and in [23]). In the stretched elliptical disks or nanowires the perturbation in *h_x_* has an insignificant effect and hence can be neglected. The *h_z_* component of the field is taken to be zero.

**Figure 1:**
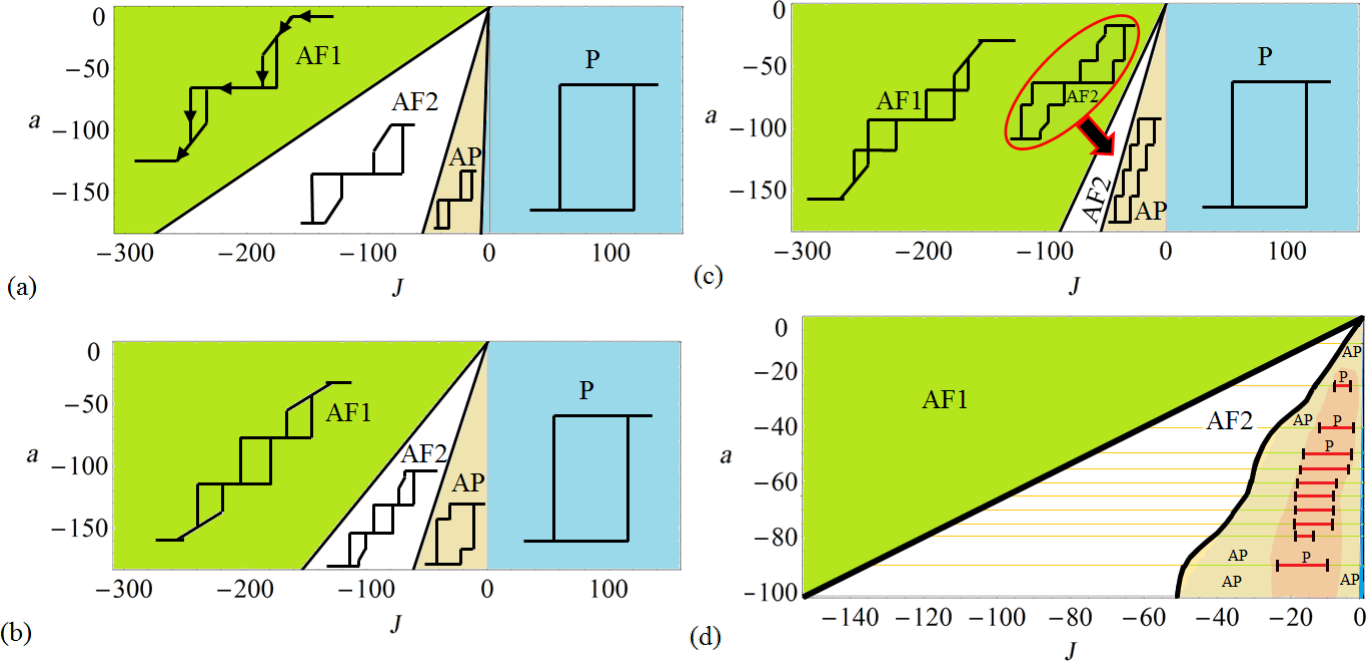
The magnetic phase diagrams of anisotropy, *a*, as a function of coupling strength *J*. In (a) *N* = 2, (b) *N* = 3, (c) *N* = 4 the phase diagrams are found through a quasi-static analysis. In (d) the dynamical results by using Equation 6 for *N* = 2 are shown. One can see that in the *AP* phase the hysteresis alternates between an *AP* and *P* phase, indicating that the *AP* phase is the least stable.

We define the phases by the shape of the magnetic hysteresis curves of magnetization as a function of the applied magnetic field strength. There are two phases involving spin-flop states: *AF*1 and *AF*2. These phases have characteristic Barkhausen jumps from scissored states into anti-parallel states of the relative orientations of the magnetic moments of each nanomagnet in a superlattice. Anti-parallel states occur when the magnetic moments point with opposite polarity along the easy-axes and are shown by a series of plateaus, in between saturation states (a parallel phase *P* occurs when there are no *AP* or *AF* states and is usually typical of ferromagnetically coupled systems). An AP phase exists when there are no scissored states. An index (*j* = 1,..) is applied to signify the number of distinct plateaus that are present in a hysteresis loop, e.g., *AF*1*_j_*. In the *AF*1 phase there is a change of state from the scissored one into a parallel one. The *AF*2 phase does not have this transition. Both the *AF*1 and *AF*2 phases, however, have transitions that go from parallel states into scissored states [15]. The *AF* phases are quite robust at the levels of damping that occur in most CoFeB systems (α ≈ 0.01). The balance between the coupling strength *J* and anisotropy parameter *a* is shown through the phase diagram of Figure 1a for the two coupled nanomagnets systems that were also considered in [15].

In this phase diagram the *AF*1 phase occurs when [15]

[8]
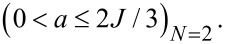


In this system the average magnetization, that evolves with the application of an external magnetic field, has a defining Barkhausen jump that emerges from an anti-parallel alignment of the magnetic moments into a scissoring spin-flop condition (the *AF*1 phase). Again, to reiterate the findings of the two nanomagnet systems [15] before increasing *N* we now state the limits of the *AF*2 phase to be,

[9]
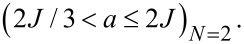


The *AP* phase exists predominantly when *a* > 2*J*. In Figure 1a the Barkhausen jump between an antiparallel state straight into a parallel one occurs, which is the nature of the *AF*2 phase. In each region of the phase diagrams of Figure 1a–c, a schematic of the shape of the magnetization versus applied magnetic field curve for the pertinent phase is shown.

The phase diagrams in Figure 1a–c are found through the use of the quasi-static techniques described in [15]. The fourth phase diagram, Figure 1d, is found from the dynamical analysis by using the LLG equations (Equation 6). From Figure 1d one can see that the *AP* phase is the least stable, with large breakdown to a *P* state within the analytically found *AP* region – compare to Figure 1a. This indicates that spin-flop phases *AF*1 and *AF*2 are more durable for logic operations. The *AP* phase, in the range of damping for CoFeB compounds around *α* = 0.01, is most stable for high anisotropies and high coupling strengths |J|. The analytically found (through the quasi-classical method [15]) phase boundary between the *AP* and *AF*2 phases holds with some degree of fluctuation for the fully dynamical analysis. However, the *AF*2–*AF*1 phase boundary corresponds completely with the analytically found solutions.

In Figure 1b, the phase diagram for three coupled nanomagnets is shown. Again there are *AF* (1,2), *AP*, and *P* regimes. When the anisotropy is in the range

[10]
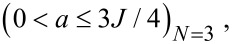


there is an *AF*1 spin-flop phase. Whereas, when the system of three nanomagnets is designed with anisotropies in the range,

[11]
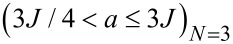


the *AF*2 phase is dominant. Beyond *a* = 3*J* the *AP* phase takes form. For *N* = 4, the phases are separated under the conditions that for the *AF*1 regime,

[12]
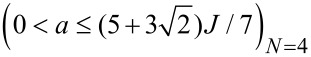


The *AF*2 phase for four layers of nanomagnets occurs in the range,

[13]
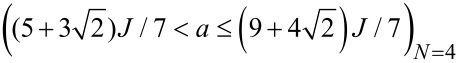


**Figure 2:**
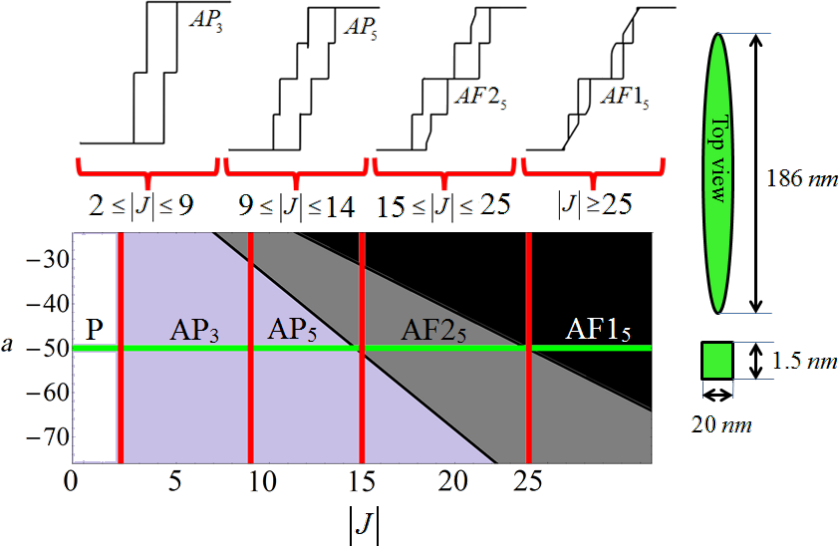
The evolution of four nanomagnets with constant anisotropy and varying interaction strength. Along the top of the plot the shape of the hysteresis, for magnetization against applied field, in a range of *J* is shown schematically. The anisotropy parameter is taken as *a* = −50 (with *N_x_* = 0.00454). The green line intersecting the bottom phase diagram represents this constant value of anisotropy. The hysteresis occurs with *j* plateaus of differing values of 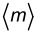, which is the meaning of the subscript of *AP_j_*, *AF*1*_j_*, and *AF*2*_j_* (plateaus of the same value of 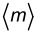 are given the same index). The hysteresis profiles have been obtained by using a full dynamical analysis using the LLG equations, Equation 6, and compared to the analytically obtained phase diagram that is similar to Figure 1. The plateaus occur at 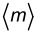 = 0, ±1/2, ±1. When 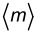 = 0, (φ_1_,φ_2_,φ_3_,φ_4_) = (0,π,0,π) or (π,0,π,0). For the saturation magnetized states, all the φ are equal. In the cases of 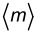 = 1/2 the azimuthal angles take the form (π,0,0,0) etc. and vice versa for 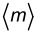 = −1/2, e.g., (π,π,π,0). Each of the nanomagnets has dimensions *l_x_*= 186 nm, *l_y_* = 20 nm, and *l_z_* = 1.5 nm. The top and front view geometry and sizes are shown schematically.

In Figure 2, the resulting hysteresis evolution as a function of |J| is shown for four coupled nanomagnets. It demonstrates that by changing the interlayer spacing and the coupling strength, completely different magnetic phases are found. Figure 2 shows the evolution of the hysteresis profile as *J* is altered. The results are obtained from a full dynamical analysis, using Equation 6, and compared to the analytically obtained phase diagram of *a* against |J| where *J* is always taken as an antiferromagnetic coupling. In Figure 2 one can see that there are clear magnetization plateaus that characterize the nature of the hysteresis (plateaus of the same value of 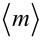 are given the same index). When 2 ≤ |J| ≤ 9, there are *j* = 3 plateaus and an *AP* phase. The dynamical analysis reveals that for very small coupling strengths, |J| ≤ 2, a *P* phase exists that has a hysteresis curve that is similar to that of a single nanomagnet in an applied field. For greater values of |J|, there are *j* = 5 plateaus in the *AP* and subsequent *AF* phases, i.e., *AP*_5_, *AF*1_5_ and *AF*2_5_.

**Figure 3:**
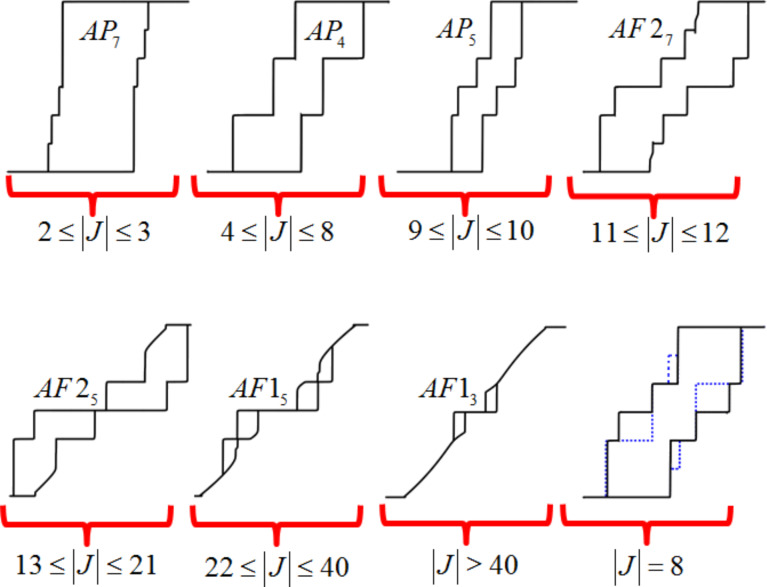
The hysteresis profiles for *N* = 6. There are fluctuating levels of magnetization plateaus as the energy balances of the system shift between metastable states. The magnetic field has to be cycled a number of times to obtain all of the possible Barkhausen jumps. For example, the last hysteresis loop shown will only display the black lines for many cycles. However, for this case of *J* = −8, further cycles reveal the dashed (blue) plateaus.

Figure 2 has four coupled nanomagnets each of dimensions *l_x_* = 186 nm, *l_y_* = 20 nm, and *l_z_* = 1.5 nm (with *N_x_* = 0.00454 and *N_z_* = 0.88269). With the inclusion of a thermally induced fluctuation, λ (see [15]) the number of plateaus can be variable. The small perturbation to the system can result in metastability and the magnetic field should be cycled many times in order to obtain all possible Barkhausen jumps, in accordance with the energy balances of the system under investigation. It is a subtle balance that exists in these energy landscapes. For example, in Figure 3 (for the anisotropy level of *a* = −50 and increasing the coupling strength steadily) there are *j* = 3–7 plateaus within the different phases. The last image in this figure shows that extra plateaus can emerge upon repeatedly cycling the magnetic field hundreds of times if the energy balance is precarious enough to allow the opportunity for finding extra metastable states. It is not, however, always the case that extra plateaus emerge. Thus, near the critical points of stability [24], at the boundaries marking phase transitions, thermally induced fluctuations in the magnetization can alter the whole nature of the magnetic evolution. Thus, the phase diagrams can be used to find the arrangement of nanomagnets that is most robust against perturbations. This is important for creating optimized computational and logic devices.

## Discussion and Conclusion

In these systems of single domain nanomagnets the macro-spin approximation holds and the internal degrees of freedom can be analyzed by using classical dynamics. We have used the dynamical Landau–Lifshitz–Gilbert equations in complement to a quasi-static analysis of the complicated energy landscapes of the interacting nanomagnets. In doing so we combine analytical solutions, obtained through quasi-classical methods [15], with numerical solutions found through a full dynamical analysis. The result is a design ethic for creating superlattice structures composed of elliptically shaped and regularly patterned magnetic particles. The quasi-static model has been used to identify possible regimes of the many particle system. Naturally the low frequency limit of the applied magnetic field must reproduce the magnetostatic approach, as it does. However we found that even in the high frequency limit, even to the gigahertz or terahertz frequency range of the applied magnetic field, that the regimes of the behavior identified within the static model still stand. The reason for this correspondence between the high and low frequency limits or static phases is due to the substantial damping, that removes the high probability for the creation of chaos in these systems. Indeed, we have found that chaos emerges within certain limits of the energy balances in the system, particularly for less elongated nanomagnets and those less damped. In our future publication we plan to address the issue of the chaos which may arise in the chains of magnetic particles. But the chaos is suppressed when the damping is suitably high, the case which we are considering here. With the emergence of new devices such as the magnetic ratchet for spintronic memory and logic in three-dimensions [25] and recent results for multisegmented nanowires [26] (where the shape anisotropy provides the preferential orientation of segmented magnetic moments along the nanowire), understanding the magnetic phases has never been more timely. Indeed, by the experimental studying of the magnetic hysteresis of these nanosized stacks the different magnetic regimes described herein can be observed. This may shed light on the complexity of the behavior of these unique and extremely interesting magnetic systems. Also, hybrid structures of ferromagnetic superlattices, combined with two-dimensional materials such as graphene and silicene have the potential to revolutionize spin-injection and detection devices for spintronics [27–30]. The ferromagnetic materials are ideal contacts for creating these spintronic devices on, for example, a single layer of graphene [29]. A magnetic ratchet device could be used to inject, at room temperature, a reservoir of spin-polarized electrons for propagation through a graphene spin barrier and avoid conductivity mismatch. The understanding of the macrospin dynamics of the superlattices can enable the design of such devices, with the spin-up/down electrons injected into the graphene for differing tunnel-transmission probabilities. Indeed, the magnetic superlattice can also be a very sensitive detector of magnetic fields, and may be used for biodetection as in [31]. Thus, these methods, discussed herein, for understanding the magnetization dynamics as a function of coupling energy and applied magnetic fields, can be used as the basis for creating novel nanomagnetic devices for magnetoresistance experiments and the creation of hybrid structures. The stability of the phases was also strongly indicated as being a function of the coupling strength between the nanomagnets for the case of a system composed of two nanomagnets. In the cases of *N* nanomagnet superlattices the phase boundaries have been highlighted by way of example by using analytical results derived from a quasi-static approach for *N* = 3 and *N* = 4. The results of the quasi-static analytics were then compared to a full dynamical analysis and it was found that a very good correspondence between them exists. Taking superlattices that are composed of *N* nanomagnets with the same geometry, i.e., *l_x_* = 186 nm, *l_y_* = 20 nm, and *l_z_* = 1.5 nm, and varying the coupling strength between them gave illuminating results. As the coupling strength is altered for larger systems of nanomagnets, *N* > 2, one can find that there are sub-phases within the main phases that are characterized by different numbers of plateaus in the hysteresis profiles. These plateaus are related to the orientations of each single-domains magnetic moments. For example, in Figure 2 there are 2*^N^* = 16 possible configurations of the magnetic moments for four coupled nanomagnets. In the hysteresis profile there are a possible *N* + 1 magnetization plateaus: those of the two saturated states, those of complete anti-parallel alignment (or with pairs of equal azimuthal angles, e.g., (0,0,π,π)), and also those when there is one nanomagnet out of phase with the rest by π radians. It was seen that for the case of four nanomagnets the *AP* phase can occur with *j* = 3 or 5 depending upon the coupling strength. As the number of nanomagnets in the superlattice is extended there exist many more metastable states and paths between energy minima in the system. In the example of Figure 3, for *N* = 6 coupled nanomagnets, this was demonstrated. In various coupling strength regimes there are sub-phases within each of the main *AF* and *AP* phases that exist with different numbers of plateaus emerging. Before a cross-over from one sub-phase into the next, e.g., Figure 3 with *J* = −8, the system is again unstable and upon cycling the applied magnetic field many times all *N* + 1 possible levels of magnetization plateaus can emerge. Thus, when designing the nanomagnetic structures, one needs to carefully investigate these sub-phases too, as the anisotropy/coupling balance strongly dictates the resulting hysteresis profile within the phase. Experiments to test for these phases and sub-phases should be designed by creating the superlattices with different inter-layer thicknesses. In doing so we hope that this will lead to greater clarity in the design process of larger, *N* > 2, arrays of nanomagnets, which will be very important in the future for creating stable magnetic devices.
